# Attitudes of consumers and live-poultry workers to central slaughtering in controlling H7N9: a cross-sectional study

**DOI:** 10.1186/s12889-017-4374-9

**Published:** 2017-05-26

**Authors:** Xiao Lin, Dingmei Zhang, Xinwei Wang, Yun Huang, Zhicheng Du, Yaming Zou, Jiahai Lu, Yuantao Hao

**Affiliations:** 1 Department of Medical Statistics and Epidemiology, School of Public Health, Sun Yat-sen University, 74 Zhongshan 2nd Rd, Guangzhou, 510080 China; 2 Sun Yat-sen Global Health Institute, Sun Yat-sen University, Guangzhou, 510080 China

**Keywords:** Attitudes, Central slaughtering policy, H7N9, Consumers, Live-poultry traders, Poultry farm workers, Infection control, Public health

## Abstract

**Background:**

Guangdong Province in the Pearl River Delta of Southeast China is among the areas in the country with the highest rates of avian flu cases. In order to control the outbreak of human-infected H7N9 cases, Guangdong launched a new policy on the central slaughtering of live poultry in 2015. This study aims to evaluate attitudes of consumers and live-poultry workers toward the policy. The live-poultry workers consisted of two sub-groups: live-poultry traders and poultry farm workers.

**Methods:**

Consumers and live-poultry workers from Guangdong were enrolled by stratified multi-stage random sampling. Online and field surveys were conducted to investigate participants’ attitudes on policy implementation. Questionnaires were developed to quantify participant demographics, to collect information about attitudes toward the policy, and to identify influential factors of policy acceptability. Proportional odds logistics regression was used in the univariate and multivariate analyses. A total of 1449 consumers, 181 live-poultry traders, and 114 poultry farm workers completed the study.

**Results:**

Policy acceptability percentages among consumers, live-poultry traders, and poultry farm workers were 57.1, 37.9, and 62.6%, respectively. Logistics regression shows that consumers tended not to support the policy if they were males, if they were concerned with the food safety of chilled products, and if they preferred purchasing live poultry. Live-poultry traders tended not to support if they were subsidized by the government, if they were males, if they experienced a drop in trading volume, and if they were unclear whether avian flu was a preventable disease. Finally, poultry farm workers tended not to support if they experienced a drop in trading volume, if they operated a poultry farm on a small to medium scale, and if they experienced inconvenience in their work due to the policy.

**Conclusions:**

The study reveals a substantial refusal or slowness to accept the policy. Failure to accept the policy results from varying reasons. Among consumers, concern about food safety and dietary preference are two major causes of disapproval. Policy acceptability among live-poultry workers diverges within the two sub-groups. While a large percentage of poultry farm workers accept the policy, the drop in trading and an insufficient subsidy hamper acceptance by live-poultry traders. We recommend that policy-makers promote health education and alleviate the policy impact on trading with a reformed subsidy policy to increase acceptability. These findings are crucial for the prevention of human-infected H7N9 cases in Guangdong.

**Electronic supplementary material:**

The online version of this article (doi:10.1186/s12889-017-4374-9) contains supplementary material, which is available to authorized users.

## Background

From 2013 to 2015, three major outbreaks of a newly-emerged avian influenza A (H7N9) virus occurred in China, creating a serious human health threat [1–3]. The first outbreak of the deadly infectious disease took place in Shanghai [1], and then occurrences spread to eastern, northern, and southern China [3] and far west areas such as Xinjiang [4]. By the end of 2015, China had reported a total of 667 H7N9 human cases with 269 deaths from over 15 provinces [5]. Most patients suffered from severe pneumonia and acute respiratory distress syndrome [6]. The clinical manifestation of H7N9 was worse than previous human H7 cases where mild/moderate cases were reported [7]. Some H7N9 cases reported a history of exposure to live poultry [8], leading to immediate suspicion of an avian reservoir as the origin of human infections. Avian-origin was further proven by Gao et al. [6] who used polymerase-chain-reaction assays, viral culturing, and sequence analyses to confirm the association between human infectious cases and reassortant H7N9 viruses. Risk factors for zoonotic H7N9 infection included physical contact with infected poultry or breathing in of contaminated aerosols/droplets [9]. Common physical contact included slaughtering, defeathering, or preparing sick poultry for cooking, handling diseased or dead poultry, and consuming raw or undercooked fowl products [10].

**Fig. 1:**
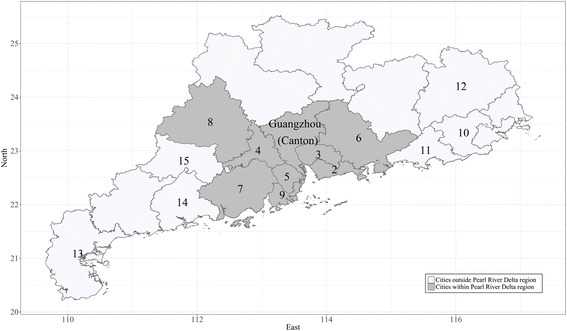
Geographical distribution of the 15 sampling cities in Guangdong, 2015. *Note:* Cities labeled by consecutive numbers ranging from 2 to 15. 2 = Shenzhen; 3 = Dongguan; 4 = Foshan; 5 = Zhongshan; 6 = Huizhou; 7 = Jiangmen; 8 = Zhaoqing; 9 = Zhuhai; 10 = Jieyang; 11 = Shanwei; 12 = Meizhou; 13 = Zhanjiang; 14 = Yangjiang; 15 = Yunfu. The map is owned by the author

This emerging avian influenza virus threatens human health in Guangdong, which is one of the provinces that have had H7N9 confirmed cases. From 2013 to 2015, Guangdong experienced severe H7N9 outbreaks, and coping strategies were launched in late 2014 after risk factors concerning the disease outbreak were studied. By the end of 2015, a total of 183 cases with 67 deaths were reported by the Health Department of Guangdong Province [11]. A risk factor of concern was the Cantonese preference for shopping at live-poultry markets for live fowl or its products [12]. Cantonese residents are the original or native-born residents in Guangdong and this population sub-group shares a similar culture, dietary habits, and social preferences when compared with populations in other parts of China [13]. Live fowl was usually slaughtered on site or brought back for home slaughter as Cantonese commonly believe that live poultry slaughtered upon purchase preserves freshness and flavour [14]. Live-poultry markets therefore play a crucial role in poultry-to-human transmission [15]. To validate this point, researchers have isolated H7N9 from environmental swab samples collected at live-poultry markets [16]. Laboratory tests then further confirmed avian influenza A virus’s ability to sustain, replicate, and disseminate at these markets [17, 18]. Clinical and epidemiological results indicated that live-poultry markets serve as a possible reservoir for avian influenza virus [15] and have placed market workers [19] as well as consumers at risk for contracting the avian influenza virus [16]. Due to the severity of H7N9 outbreaks in 2013–2014, the local preference for purchasing live fowl, and the high risk of virus exposure, the government of Guangdong Province announced a new policy on the central slaughtering of live poultry in late 2014. The Central Slaughtering of Live Poultry Policy (CSLPP) was officially set to take effect in the 21 cities in Guangdong Province on January 15, 2015 [20]. The CSLPP is a comprehensive policy combining several previously-proven biosecurity measures with some unconfirmed measures. Though policy regulations are consistent across the entire province, their implementation is determined by prefecture-level governments. Implementation priorities are given to the cities within the Pearl River Delta region to set ‘Leadership Cities’ for the whole province (Fig. 1). However, broader knowledge of influential factors of attitudes toward policy is lacking at prefecture levels and thus it is important to address these issues before further implementation of the CSLPP is carried out. General information on the CSLPP can be found in Table 1 and on the government website [20].

**Table 1:** Key Elements of Central Slaughtering of Live Poultry Policy

• Creation of live-poultry-restricted areas where new wholesale poultry markets cannot be built and live-poultry trading is restricted to only a few qualified retail/wholesale markets
• Regulations on central processing of live poultry in only a limited number of licensed slaughterhouses within live-poultry-restricted areas
• Ban on taking live poultry out of wholesale/retail markets within live-poultry-restricted areas
• Ban on trading live poultry outside live-poultry markets that are within live-poultry-restricted areas
• Ban on leaving live fowl overnight at live-poultry markets within live-poultry-restricted areas [33]
• Regulations related to daily disinfection procedures at live-poultry markets within
• live-poultry-restricted areas [34] and weekly cleansing routines
• Closure of live-poultry markets within live-poultry-restricted areas on a monthly basis [35]
• Regulations on handling diseased poultry and potential contaminated facets
• Segregation of live fowl from other market products, and creation of physical barriers between consumers and poultry holding, slaughter, or sale zones
• Requirement to teach essential basics on prevention of the avian influenza infection among poultry market workers
• Installation of refrigerators and containers to preserve chilled products [extra expenses for installation covered by government subsidies]
• Regulations on transportation delivery of chilled products to markets

Because the CSLPP is a new policy, questions still need to be answered on perceptions and attitudes of this policy among general consumers and poultry workers. Although a previous paper assessed consumers’ attitudes toward central slaughtering, its findings were confined to the city of Guangzhou [21], and further work is needed to determine if those findings can be extrapolated to other parts of Guangdong Province. Also, influential factors of public acceptance of the CSLPP require further study for the successful long-term implementation of the policy. Therefore, the current study aims to assess and better understand the acceptability of the CSLPP and its influential factors among consumers and live-poultry workers on a larger scale in Guangdong Province.

## Methods

This study is a cross-sectional observation in assessment of attitudes among consumers and live-poultry workers toward the CSLPP, conducted from October to November, 2015. Live-poultry workers are sub-grouped into live-poultry traders and poultry farm workers. Stratified three-stage random sampling and online/field recruitment were employed in sampling of participants (Additional file 1: Table S1). The 21 prefectural-level cities in Guangdong were stratified into cities that are located in the Pearl River Delta region and those that are not. A total of 15 cities were randomly selected using the random number method in the first stage. Live-poultry markets and live-poultry farms were randomly selected using the random number method in the second stage. Participants were then randomly selected in the final stage. We designed different questionnaires to survey attitudes toward the CSLPP among consumers and the two sub-groups of live-poultry workers. Interviewers were recruited and trained to comply with uniform survey protocol so that the quality of the survey was ensured. We followed the guidelines of the STROBE (STrengthening the Reporting of OBservational studies in Epidemiology) Statement in this paper.

### Sample collection

#### Sampling of consumers

We defined the population of consumers as those who lived in Guangdong and had once purchased or consumed live-poultry products. Consumers of live-poultry products living in the 15 cities were recruited as participants for the study. In sampling consumers, we adopted both field and online recruitment (Additional file 1: Table S1). Field surveys were carried out in Guangzhou, Foshan, and Shenzhen, where we deployed our trained interviewers at market entrances and randomly selected consumers as they entered. The markets were also randomly selected. Consumers were asked if they or their family had purchased live poultry before (screening question). We further investigated consumers who gave a positive reply and expressed willingness to participate in the survey. To recruit consumers in other cities, notices about the questionnaire were posted on WeChat, including a link to an external survey website where participants were able to click on and fill out the questionnaire. The trained interviewers used the same screening question above to identify potential participants in their chat-groups and randomly selected target participants. They sent WeChat notices to target participants. Interviewers also provided online survey instructions to participants to ensure the validity of the online surveys. In the end, 1506 consumers completed questionnaires, but only 1449 (96.2%) consumers lived in the selected cities and were thus considered appropriate for analysis.

#### Sampling of live-poultry workers

We defined the population of live-poultry workers as those who worked either at live-poultry markets or poultry farms in Guangdong. Live-poultry workers either at live-poultry markets or poultry farms in the 15 cities were recruited as participants for the study. Field surveys were conducted in the investigation of live-poultry workers. For different sub-groups of live-poultry workers, we conducted random sampling in different locations. Live-poultry traders were sampled and surveyed at live-poultry markets, while poultry farm workers were surveyed at poultry farms. Live-poultry markets and poultry farms were randomly selected in each city. We approached all live-poultry traders and poultry farm workers at each live-poultry market and poultry farm. A quick on-site review of the questionnaires was conducted to ensure data validity. A total of 181 (100.0%) live-poultry traders and 114 (100.0%) poultry farm workers completed the questionnaires. (All 181 and 114 samples are considered appropriate for data analysis because the enrollment requirement for each sample was satisfied.) Additional information on sampling strategies and power analysis can be found in Additional file 1: Table S2.

### Measures

A separate questionnaire was developed for live-poultry farm workers, live-poultry traders and consumers of live-poultry products (Questionnaires are provided in Additional file Q1, Q2, Q3). The questionnaires were designed after review of the policy and previous research [14, 22]. We conducted a pilot survey in the city of Guangzhou and also consulted field experts to ensure the validity of the questionnaires.

All questionnaires covered a range of four dimensions. The first dimension began with demographic questions, such as age, sex, and local resident status of the respondent. Other questions, depending on their respondent type, were included for background information on their experience with live poultry. The second dimension evaluated knowledge and perceptions of avian flu. We converted choice-results into scores where each correct answer was counted as one point for a total score of 12. The third dimension explored the perceptions of the CSLPP including whether they had heard of the CSLPP and the source of their hearing about the policy. Other questions, depending on their respondent type, were included for background information on their perception with the CSLPP. The final dimension assessed influential factors on attitudes toward the CSLPP. The whether-or-not questions were either binary (yes/no) or ternary (yes/no/no idea). (Additional file 1: Table S3) Attitude item was a three-level ordinal outcome variable. Disapproval of the CSLPP was the lowest level, indifference to the policy the middle, and acceptability the highest.

### Statistical analysis

Descriptive statistics were derived from each questionnaire. Consumers and live-poultry workers were analysed separately. Poultry traders and farm workers were analysed as a group, with farm worker and trader status being included as a possible explanatory variable. The sum-up score of knowledge on avian flu was converted into a three-level knowledge variable. A chi-squared test was used in the univariate analyses to compare the indicators between regions. Proportional odds logistics regressions were conducted in the univariate and multivariate analyses to explore contributions of demographic variables, attitude variables, and knowledge variables to acceptability of the CSLPP. Initial variables for model selection were listed in Additional file 1: Tables S6 and S7, and final variables were selected based on Akaike information criterion (AIC). Odds ratio (OR) lower than one indicated disapproval of the policy. All statistical analyses were computed by R version 3.2.3, and *P* < 0.05 indicated statistical significance.

## Results

### Sociodemographic characteristics of participants.

The sociodemographic characteristics of participants are listed in Table 2. Sampled consumers and live-poultry workers had an average age of 25 years (SD 13.0) and 41 years (SD 9.0), respectively. For consumers sampled, over 30% resided in Guangzhou and 77.5% lived in the Pearl River Delta region, but most reported being native-born Cantonese (80.0%). As for the sub-groups of live-poultry workers, most live-poultry traders sold chicken (97.2%) and ducks (84.0%) and their sales volume in median was 30 per day. Over a half (64.8%) of the live-poultry traders surveyed worked in the Pearl River Delta region. Most poultry farm workers bred chicken for commercial trading (89.6%) and some bred ducks (10.4%). Also, participating poultry farm workers reported that their cities had once reported confirmed cases of the avian influenza virus (36.8%), and that their farms had a trade volume of 100,000 or more per year (77.0%) and a business history of over 5 years (58.9%). A total of 64.7% of surveyed farm workers worked in the non-Pearl River Delta region.

**Table 2:** Sociodemographic characteristics of surveyed participants from 15 cities, Guangdong, China, 2015

	Sum-up *N* (%)	Pearl River Delta *N* (%)	Non-Pearl River Delta *N* (%)
Consumers (*n* = 1449)			
Sample	1449 (100.0)	1123 (77.5)	326 (22.5)
Gender**			
Male	579 (40.0)	424 (37.8)	155 (47.5)
Female	870 (60.0)	699 (62.2)	171 (52.5)
Age**			
15 ~ 20	694 (47.9)	518 (46.1)	176 (54.0)
21 ~ 30	316 (21.8)	246 (21.9)	70 (21.5)
31 ~ 40	250 (17.3)	205 (18.3)	45 (13.8)
41 ~ 50	126 (8.7)	95 (8.5)	31 (9.5)
≥ 51	63 (4.3)	59 (5.3)	4 (1.2)
Mean (SD)	25 (13.0)	26 (13.0)	23 (11.0)
Education			
Primary or below	23 (1.6)	22 (2.0)	1 (0.3)
Secondary	83 (5.7)	63 (5.6)	20 (6.1)
High School	232 (16.0)	179 (15.9)	53 (16.3)
Tertiary or above	1111 (76.7)	859 (76.5)	252 (77.3)
Income (Yuan per capita/month)**			
< 2000	37 (2.6)	28 (2.5)	9 (2.8)
2000 ~ 2999	76 (5.2)	56 (5.0)	20 (6.1)
3000 ~ 3999	216 (14.9)	155 (13.8)	61 (18.7)
4000 ~ 4999	258 (17.8)	176 (15.7)	82 (25.2)
5000 ~ 5999	190 (13.1)	136 (12.1)	54 (16.6)
≥ 6000	164 (11.3)	121 (10.8)	43 (13.2)
Local Cantonese**			
Yes	1159 (80.0)	849 (75.6)	310 (95.1)
No	290 (20.0)	274 (24.4)	16 (4.9)
Live-poultry workers (*n* = 295)			
Sample	295 (100.0)	261 (88.5)	34 (11.5)
Gender**			
Male	180 (61.0)	167 (64.0)	13 (38.2)
Female	115 (39.0)	94 (36.0)	21 (61.8)
Age^a^			
17–30	44 (15.1)	41 (15.9)	3 (8.8)
31–40	88 (30.1)	71 (27.5)	17 (50.0)
41–50	119 (40.8)	106 (41.1)	13 (38.2)
≥ 51	41 (14.0)	40 (15.5)	1 (2.9)
Mean (SD)	41 (9.0)	42 (9.0)	39 (6.0)
Employment status			
Employee	136 (46.1)	126 (48.3)	10 (29.4)
Employer	159 (53.9)	135 (51.7)	24 (70.6)
Classification of occupation**			
Live-poultry traders	181 (61.4)	169 (64.8)	12 (35.3)
Poultry farm workers	114 (38.6)	92 (35.2)	22 (64.7)

^a^Statistical significance was found in group comparison between items in the Pearl River Delta and those outside the Pearl River Delta using the method of the chi-squared test. Age distribution of live-poultry workers from the Pearl River Delta was statistically different from that of those outside the Pearl River Delta (*P* = 0.02)

**P value was equal to or lower than 0.01. The chi-squared test was used in the statistical analysis. Gender distribution was statistically different for consumers living in the Pearl River Delta region and those outside the region (*P* = 0.002). Age distribution was statistically different for consumers living in the Pearl River Delta region and those living outside the region (*P* = 0.003). Income status and the status of being a Cantonese were statistically different between regions (both *P* values were lower than 0.001). Gender distribution was statistically different for live-poultry workers in the Pearl River Delta region and outside the region (*P* = 0.007). Live-poultry traders and poultry farm workers were statistically different between regions (*P* = 0.002)

### Perception and acceptability of the CSLPP.

This study found that 58.5% of consumers knew about the CSLPP. (Additional file 1: Table S4) Among those who had heard of the policy, 57.1% expressed support for the policy, 26.5% were indifferent to the policy, and 16.4% disapproved of the policy. This study also summarized policy acceptability of consumers across cities. (Additional file 1: Table S5) Over half (52.4%) of consumers in Guangzhou supported the policy. Univariate analysis of concerning factors (Additional file 1: Table S6) showed that consumers who raised an objection to the CSLPP might possess the following traits: native-born Cantonese, (family) preference to purchase live poultry slaughtered on site, not believing in the food safety of chilled poultry products, etc.

For live-poultry workers, while most knew about the policy (92.6%) and also understood its purpose (96.7%), acceptability differed within sub-groups. For live-poultry traders, 37.9% supported the policy but 45.4% disapproved of the policy. For poultry farm workers, 62.6% supported the policy and 11.1% disapproved of the policy. The remaining were indifferent to the policy (16.6% and 26.3% for live-poultry traders and poultry farm workers, respectively). A total of 69.5% of live-poultry traders complained about the inconvenience caused by the CSLPP where only 24.2% of poultry farm workers complained about inconvenience (Table 3). Univariate analysis was used to explore lower acceptability among live-poultry traders, and significant factors include: being an employer, loss in trading volume, given subsidies, inconvenience induced by the CSLPP, and low scores on knowledge of avian flu, etc. (Additional file 1: Table S7).

**Table 3:** Perception related to the CSLPP^a^ among sub-groups of live-poultry workers from Guangdong, China, 2015

Questionnaire Items	Yes *N* (%)	No *N* (%)
Live-poultry traders		
Heard of the Central Slaughtering of Live Poultry Policy (CSLPP)	174 (96.1)	7 (3.9)
Knew the purpose of the CSLPP (to control avian flu)	166 (95.4)	8 (4.6)
Drop in trading volume after launch of the CSLPP	153 (87.9)	21 (12.1)
Rise of expense after launch of the CSLPP^b^	134 (77.0)	40 (23.0)
Subsidies given by government	94 (54.0)	80 (46.0)
Whether subsidies are enough or not	39 (41.9)	54 (58.1)
Detail of the CSLPP ever explained by market managers	157 (90.2)	17 (9.8)
Belief in effectiveness of the CSLPP in controlling avian flu	108 (62.1)	66 (37.9)
Belief in enhancement of environment as a result of the CSLPP	143 (82.2)	31 (17.8)
Inconvenience to work induced by the CSLPP	121 (69.5)	53 (30.5)
Convenience to work induced by the CSLPP	49 (28.2)	125 (71.8)
Acceptability of the policy	66 (37.9)	79 (45.4)
Poultry farm workers		
Heard of the CSLPP	99 (86.8)	15 (13.2)
Knew the purpose of the CSLPP (to control avian flu)	98 (99.0)	1 (1.0)
Drop in trading volume after launch of the CSLPP^c^	60 (60.6)	39 (39.4)
Detail of the CSLPP ever explained by government workers	76 (76.8)	23 (23.2)
Belief in effectiveness of the CSLPP in control of avian flu	83 (83.8)	16 (16.2)
Belief in enhancement of environment as a result of the CSLPP	83 (83.8)	16 (16.2)
Inconvenience to work induced by CSLPP	24 (24.2)	75 (75.8)
Convenience to work induced by CSLPP	43 (43.4)	56 (56.6)
Acceptability of the policy	62 (62.6)	11 (11.1)

^a^ *CSLPP* Central Slaughtering of Live Poultry Policy

^b^A total 86.2% of live-poultry traders reported a drop in profit after the launch of the CSLPP (11.5% profit unchanged, 2.3% profit increased)

^c^A total of 53.5% of poultry farm workers reported a drop in profit after the launch of the CSLPP, but 39.4% reported profit being unchanged

### Multivariate analysis for policy acceptability.

To further explore the influential factors of acceptability toward the policy in the previous univariate analysis, multivariate analyses were conducted among participating consumers and sub-groups of live-poultry workers. Table 4 shows the variables in the final proportional odds logistics regression models. In the consumer model, 15 variables were selected and the rest were screened out based on AIC values of the models. Some major influential variables leading to disapproval of the policy include: being a male, being a native Cantonese, preferring live poultry, etc. In the model for live-poultry traders, six variables entered the model. Live-poultry traders tended to disapprove of the policy if they: a) were males; b) were subsidized by the government; c) experienced a drop in trading volume; and d) were unclear whether avian flu was a preventable disease. Finally, poultry farm workers tended not to support if they: a) experienced a drop in trading volume; b) operated a poultry farm on a small to medium scale, and c) experienced inconvenience to their work due to the policy.

**Table 4:** Multivariate Logistics models for influential factors of acceptability toward CSLPP^a^

Variables	Values	β	Std. Error	OR (95% CI)
Consumers				
Gender	Male	−0.3	0.2	0.8 (0.6–1.0)
Native Cantonese	Yes	−0.7	0.2	0.5 (0.3–0.8)
Preference of purchasing live poultry	Yes	−0.5	0.2	0.6 (0.4–1.0)
Which tastes best	Defeathered	−0.1	0.3	0.9 (0.5–1.6)
	Chilled	−0.3	0.6	0.8 (0.3–2.5)
	Frozen	−0.4	1.3	0.7 (0.1–17.1)
	No difference	1.0	0.3	2.8 (1.5–5.5)
More convenient to buy chilled product	No	−0.8	0.2	0.5 (0.3–0.7)
Media propaganda on purchase	No	−0.6	0.2	0.6 (0.4–0.8)
Nearby live-poultry retail store	Yes	0.7	0.2	1.9 (1.3–3.0)
	No	0.6	0.3	1.8 (1.1–3.0)
Belief in food safety for live-poultry product	No	−0.5	0.2	0.6 (0.5–0.9)
Prevention of avian influenza	No	−1.1	0.2	0.3 (0.2–0.5)
Enhancement of environment	No	−1.5	0.3	0.2 (0.1–0.4)
Frequency of purchasing	Increase	1.4	0.4	3.8 (1.7–9.8)
	Decrease	−0.7	0.2	0.5 (0.4–0.7)
Which type purchased most	Defeathered	0.6	0.2	1.7 (1.2–2.5)
	Chilled	0.5	0.3	1.7 (1.0–2.8)
	Frozen	1.3	1.2	3.8 (0.4–82.2)
The avian influenza to be a serious disease	Yes	1.1	0.4	2.9 (1.3–6.5)
	No	0.6	0.4	1.8 (0.8–4.2)
Wearing hand-gloves	Yes	0.3	0.2	1.4 (1.0–1.9)
Handwashing	Yes	0.7	0.4	2.1 (1.0–4.2)
Live-poultry traders				
Gender	Male	−0.7	0.3	0.5 (0.3–0.9)
Drop in trading volume	Yes	−1.2	0.6	0.3 (0.1–0.9)
Subsidized by the government	Yes	−0.7	0.3	0.5 (0.3–1.0)
Policy explained by market managers	Yes	−1.0	0.6	0.4 (0.1–1.1)
Belief in effectiveness of the CSLPP	For prevention of avian flu	0.7	0.4	2.0 (1.0–4.1)
	For enhancement of environment	0.8	0.5	2.3 (0.9–5.8)
Considered avian flu to be a preventable disease	No idea	−1.7	0.5	0.2 (0.1–0.5)
	No	0.2	0.6	1.2 (0.4–4.3)
Poultry farm workers				
Age	≤30	−0.3	1.1	0.8 (0.1–6.7)
	31 ~ 40	2.0	1.0	7.4 (1.1–62.4)
	41 ~ 50	0.6	1.0	1.8 (0.3–13.0)
Employment status	Employer	3.0	1.1	20 (2.9–207.9)
Farm scale	Small	−2.4	1.0	0.1 (0.01–0.6)
	Medium	−2.2	0.8	0.1 (0.02–0.6)
Drop in trading volume	Yes	−2.4	1.1	0.1 (0.01–0.7)
Policy explained by government workers	Yes	1.3	0.7	3.6 (1.0–14.7)
Belief in effectiveness of the CSLPP	For enhancement of environment	1.5	0.7	4.3 (1.1–20.3)
Inconvenience to work resulted from the policy	Yes	−2.0	0.7	0.1 (0.03–0.5)
Convenience to work resulted from the policy	No	−1.6	0.8	0.2 (0.04–1.0)

^a^ *CSLPP* Central Slaughtering of Live Poultry Policy

## Discussion

To the best of our knowledge, this paper is the first study that employed so large a sample to evaluate the attitudes to and acceptability of the CSLPP among consumers in Guangdong Province. The study’s large sample of consumers and sampling strategies help ensure the representativeness of the sample. The large sample provides a 93% statistical power for data analysis and such a high power should suggest that the sample is large enough for subsequent statistical analysis and the corresponding statistical results are valid and robust. (Additional file 1: Table S2) Sampling strategies include enrollment requirements and imposing randomization. We mainly targeted the population of consumers as those who live in Guangdong and have purchased or consumed live-poultry products. Participants satisfied an enrollment requirement through surveys: in the field survey, we differentiated potential consumers at live-poultry markets from passers-by and identified consumers of interest by asking the screening question; in the online survey, we identified consumers of interest by the screening question. We imposed randomization on the selection of cities, the selection of live-poultry-restricted areas, and the selection of live-poultry markets. While this is the first research study to employ such a large sample of consumer reactions to the CSLPP in Guangdong Province, it is also the first to address attitudes toward the CSLPP among consumers and sub-groups of live-poultry workers in mainland China. The CSLPP has had proven-success in controlling avian influenza outbreaks [10], but although the policy appears to be a practical and seemingly cost-effective way to help control avian influenza outbreaks, a substantial portion of the public may think otherwise. According to our study, over half of consumers surveyed supported the policy, but 45.4% of live-poultry traders expressed strong objections to it. Low acceptability may hamper the effectiveness of the policy and challenge the campaign against avian flu; thus, identifying and understanding the factors that influence negative acceptability is especially important. Our study further explores influential factors of acceptability among surveyed groups by quantitative methods. For instance, one result of the study indicates that the concern over food safety of chilled products and the preference for purchasing live poultry comprise an influential factor for policy acceptability (Table 4), a finding corroborated by a previous study led by Wen et al. [14] where it was concluded that a) the concern of loss of freshness would result in not buying chilled poultry products; b) some Cantonese believe the flavor and freshness of poultry can be best preserved if slaughtered on-site; and c) some believe on-site slaughtered poultry products can guarantee food safety. We consider that long-held local beliefs and biases and a lack of scientific proof for better methods have led to the difficulty of changing the millennium-long practice of trading live poultry and the habit of consuming freshly killed-and-cooked poultry [12]. Through reduction of poultry availability and an increase in health education messages, however, these practices and habits may be changed, as we have seen in Hong Kong where residents recognized the policy’s positive effects in helping control the risk for avian flu [23]. Our study results indicate that policy acceptability among consumers in Guangzhou in 2015 was slightly higher than what Yuan et al. reported in early 2014 [21]. The slight rise of acceptability in only 2 years is possibly due to Guangdong’s efforts at prevention [20], including use of media publicity about the CSLPP and educating the public on avian flu prevention.

While the extent to which these efforts have helped raise acceptability is unknown and future investigation is warranted, our survey indicates the possibility of raising acceptability by planning for long-term health education and media publicity. As stated, however, almost half of live-poultry traders surveyed disapproved of the CSLPP. The data in our survey on this group is based on samples noticeably smaller than that of consumers, but we believe that the opinions on the CSLPP of live-poultry workers in our random sample represent those of the total population of live-poultry workers in Guangdong, given the typical small size of the total population of live-poultry workers in a city, as indicated by the China consensus report [24]. In the live-poultry traders sub-group, the proportion of live-poultry traders among all market traders is estimated to be small: in fact, our interviewers reported that each market may house no more than five poultry traders. (Additional file 1: Table S2) The situation is similar for poultry farms. Not every city has live-poultry farms. Many poultry farms are being closed especially in the Pearl River Delta region, as with the city of Shenzhen which closed most of theirs and imported chilled products from other cities [25]. Thus, the unbalanced sample size accounts for the unbalanced distribution of poultry farms; and the lack of balance in the sample sizes of both sub-groups of live-poultry workers accounts for the relative sizes of these populations. Sample strategies we employ help ensure sample representativeness as well. We surveyed as many live-poultry traders as possible for each randomly-selected live-poultry market and all poultry farm workers, within a randomly-selected live-poultry-restricted area in each city for each representative region. (Additional file 1: Table S2) The unequal geographical distribution of the sample of live-poultry workers should not be a drawback but a merit in the study. The CSLPP was initially piloted in the cities of Guangzhou, Foshan, and Shenzhen in 2014 [26], all of which are in the Pearl River Delta region. After this, all other cities in the Pearl River Delta region followed and finally the cities outside the Pearl River Delta. The extent to which the policy was implemented differs between the two regions, but it is due to the differences between them that Pearl River Delta cities became ‘Leadership Cities’ to best represent Guangdong in terms of policy implementation. The sampling strategy thus gave a greater stratum to the cities within the Pearl River Delta region, and the sample proportion is larger for Pearl River Delta cities. Thus, our random sample of live-poultry workers is reflective of actual population proportions, distribution, and implementation demographics, and for these reasons we believe that it is representative of the total population of live-poultry workers in Guangdong Province.

As revealed in this study, opinions toward the CSLPP among live-poultry workers diverges. The divergence is intriguing, and our study focuses on exploring the reasons particularly behind the low acceptability among live-poultry traders. Table 3 indicates that characteristics concerning perception and acceptability of the CSLPP varied within different sub-groups of live-poultry workers. Statistical analyses are conducted separately for each sub-group. Revealed by univariate and multivariate analyses, one of the most influential factors is the decline in trading volume. Indeed, a previous paper roughly estimated a loss of 57 billion yuan (about US $8 billion) due to closures of live-poultry markets [27]; for poultry farm workers, the drop in trading volume would seem to sufficiently explain their disapproval of the policy. We project, though, that the gains of the CSLPP will eventually transcend this financial loss if it becomes a long-term regulation or rule, especially given a concomitant change in consumer attitudes. The fluctuating trade volume before and after the implementation of the CSLPP may be heavily influenced by consumers since their preferences and purchases have such a great impact on the supply and demand chain. If consumers accept the changes, the market and live-poultry workers may also. Thus, to promote acceptability among live-poultry traders, we suggest that the government place emphasis on further transforming consumers’ attitudes and habits. Also, further cost-effective analysis should be conducted to ascertain the actual validity of the CSLPP. Perceived insufficiency of the government subsidy is another influential factor. The perception of an insufficient subsidy hampered acceptability as shown in the univariate (Additional file 1: Table S6) and multivariate analysis (Table 4). This finding is rather interesting as it might oppose government projected outcomes. From the dataset, it appears that 58.1% of live-poultry traders thought the government subsidy was insufficient (Table 3), regardless of the fact that the subsidy already covered the expenses for installation of refrigerators and containers. This comprises a major cause of low acceptability. Beyond the dataset, then, we believe that more work is needed to study the low acceptability induced by the perception of an insufficient government subsidy.

Our study has several limitations. First, we did not collect information on respondents that were approached but refused to participate. Therefore, we were unable to exactly calculate a survey response or refusal rate, as the information was not collected. Nonetheless, we believe that we have over-estimated the acceptability rate, especially for the case of live-poultry traders, based on the findings of a market survey conducted in the cities of Guangzhou, Shenzhen, Dongguan, Foshan and Yangjiang [28]. It has been reported by Tan et al. [28] that each live-poultry market may house no more than five live-poultry traders. Based on their findings, we deduce that the response rate of live-poultry traders should be around 70% (Additional file 1: Table S2) and the rejection-weighted acceptability rate of CSLPP among live-poultry traders should be around 26.4%. The even lower acceptability among live-poultry traders should nonetheless suggest that their reluctance to accept the CSLPP remains an obstacle to the successful implementation of the policy. Further implementation of the policy should thus take into account some positive intervention that help change negative points of view among live-poultry traders. Second, the age distribution of the consumer sample is shifted to the younger age group. The fact that consumers in some cities were sampled in person while consumers in other cities were sampled via internet self-selection methods should account for the shift. The online survey is known to be popular among the younger population [29, 30] and widely used in the era of Internet technology [31, 32]. As a consequence, the older population may be screened out by this survey method and selection bias introduced. The fact that we used WeChat as the survey platform and its younger-age-shifted user groups [30] explains our shifted sample of consumers. However, in the study, we did not incorporate age limitation in the definition of consumer population. Therefore, even though participants may be younger, they should still be regarded as consumers and their opinions on the policy should also be respected. In other words, the age effect should not be a factor of concern for the interpretation of the results. Nonetheless, the results in the study should at least account for what younger consumers think of the CSLPP. The online survey may also have a geographic effect in which participants outside the 15 cities selected could have completed the questionnaire. However, as stated above, the participants living outside the selected cities were dropped out and a total number of 1449 consumers remained in the final dataset for analysis.

### Policy implications

When compared with investment in developing vaccines for avian flu viruses and expenses in treatment for severe avian flu cases, it seems that the implementation of the CSLPP is a much more economical method as it cuts the transmission route of the virus, although a detailed cost analysis would be needed to determine the extent to which it helps save money in the campaign for the prevention of the avian influenza outbreaks. We suggest that policy acceptability could be increased and that implementation of central slaughtering could be enhanced with the following strategies.

#### Enhance the promotion of the CSLPP

The government should continue to disseminate the robust detail and positive influence of the CSLPP via public-welcomed methods including TV shows, magazine articles and advertisements, and community billboards. Alternatively, people should be educated about the cold-chains for chilled products and be taught about the procedures that help prevent pre-processed meats from bacterial infection. Through these efforts, people’s uninformed opinions on chilled products may undergo a change, and they may realize the significance of the policy to their health.

#### Improve subsidy policy for implementation of the CSLPP

A reform of the subsidy policy and enhancement of the satisfaction of live-poultry traders should become an important agenda. Extra support should be given to live-poultry workers to cover the potential loss of customers and drop in profits. Bonuses should also be granted to live-poultry workers for their contribution to implementation of the CSLPP. Also, both provincial and prefecture governments should rethink the strategy of fiscal allocation so that a subsidy system could become a government-run insurance and support system for live-poultry traders in the long run.

#### Take steps in implementation of the CSLPP accordingly but seize the opportunity to educate the public

The policy-maker should never expect quick results from the CSLPP, and special attention should be given to the areas where public objection to the CSLPP is strong. To deal with objections, we suggest small trial zones be set up. In these small trial zones, the government should practice the CSLPP in several steps. Local residents should be given the time that is needed to adjust their attitudes and behaviors. But it is also important that they be educated about the flu, most especially during times of outbreak, so that the effect of education and promotion can be strengthened.

## Conclusions

This study concludes that acceptability of the policy among live-poultry traders is low and divergent within sub-groups of live-poultry workers. Possible reasons for low acceptability among live-poultry traders include: the drop in trading volume, the insufficient subsidy offered by the government, and the misconception about avian flu. Concern about food safety and dietary preference are two major reasons for disapproval of the policy among consumers. These findings are crucial to the prevention of human-infected H7N9 cases in Guangdong Province.

## Additional file

Additional file 1: Table S1. Summarizes the survey protocols. **Table S2.** shows detailed information on the enrolled cities, live-poultry-restricted areas and sample size for each survey. **Table S3.** shows attitudes and preventive behaviors among consumers and live-poultry workers. **Table S4.** shows perception of the policy among consumers. **Table S5.** shows acceptability rate of the policy among consumers in different cities selected. **Table S6.** shows results of univariate analysis for policy acceptability among consumers. **Table S7.** shows results of univariate analysis for policy acceptability among live-poultry workers. **Q1.** shows the questionnaire used for investigating consumers. **Q2.** shows the questionnaire used for investigating live-poultry traders. **Q3.** shows the questionnaire used for investigating poultry farm workers (DOCX 90 kb).

## Abbreviations

AIC: Akaike information criterion
CI: 95% confidence interval
CSLPP: Central Slaughtering of Live Poultry Policy
OR: Adjusted odds ratio
SD: Standard deviation
STROBE: STrengthening the Reporting of OBservational studies in Epidemiology
