# Supplementary material for: Attitudes of consumers and live-poultry workers to central slaughtering in controlling H7N9: a cross-sectional study
Source: BMC Public Health. 2017 May 26;17:517. doi: 10.1186/s12889-017-4374-9 (PMC5446744; doi:10.1186/s12889-017-4374-9)
Supplement: Additional file 1: Table S1. — Summarizes the survey protocols. Table S2. shows detailed information on the enrolled cities, live-poultry-restricted areas and sample size for each survey. Table S3. shows attitudes and preventive behaviors among consumers and live-poultry workers. Table S4. shows perception of the policy among consumers. Table S5. shows acceptability rate of the policy among consumers in different cities selected. Table S6. shows results of univariate analysis for policy acceptability among consumers. Table S7. shows results of univariate analysis for policy acceptability among live-poultry workers. Q1. shows the questionnaire used for investigating consumers. Q2. shows the questionnaire used for investigating live-poultry traders. Q3. shows the questionnaire used for investigating poultry farm workers (DOCX 90 kb). [file 12889_2017_4374_MOESM1_ESM.docx]

Attitudes of consumers and live-poultry workers to central slaughtering in controlling H7N9: A cross-sectional study

**Xiao Lin^1^, Dingmei Zhang^1^, Xinwei Wang^1^, Yun Huang^1^, Zhicheng Du^1^, Yaming Zou^1^, Jiahai Lu^1^, Yuantao Hao^1,2,*^**

^1^Department of Medical Statistics and Epidemiology, School of Public Health, Sun Yat-sen University, Guangzhou, 510080, China.

^2^Sun Yat-sen Global Health Institute, Sun Yat-sen University, Guangzhou, 510080, China.

Contents

**Additional file 1: Table S13**

**Additional file 1: Table S24**

**Additional file 1: Table S36**

**Additional file 1: Table S47**

**Additional file 1: Table S59**

**Additional file 1: Table S610**

**Additional file 1: Table S712**

**Additional file 1: Q115**

**Additional file 1: Q217**

**Additional file 1: Q319**

**Reference21**

**Additional file 1: Table S1.** Summary of the survey protocols

| Category | Survey type | Survey method | Survey topics (dimensions) |
| --- | --- | --- | --- |
| Consumers^†^ | Questionnaire with closed ending questions | Both self-submit online questionnaire and self-administered on-site questionnaire, with supervision and instruction | Demographical characteristics (age, gender,…)  Perception and acceptability of the CSLPP**^†††^**  Perception of avian disease and related behaviour |
| Live-poultry traders | Questionnaire with open- ending questions | On-site interviewer-administered questionnaire | Demographical characteristics (age, gender,…)  Trading volumes, expenses and profits  Subsidies and convenience  Perception and acceptability of the CSLPP  Perception of avian disease and related behaviour |
| Poultry farm workers | Questionnaire with open- ending questions | On-site interviewer-administered questionnaire | Demographical characteristics (age, gender,…)  Trading volumes and convenience  Perception and acceptability of the CSLPP  Perception of avian disease and related behaviour |

^†^Participant recruited online was considered to be a consumer of poultry product if he/she or his/her family had once purchased such product.

^†††^CSLPP=Central slaughtering of live poultry policy.

**Additional file 1: Table S2.** Enrolled cities and detail on live-poultry-restricted areas and sample size for each survey

| Enrolled city | Number of live-poultry-restricted areas/Total number of live-poultry-restricted areas^†^ | No. consumers. surveyed | No. live-poultry traders. surveyed | No. poultry farm workers. surveyed |
| --- | --- | --- | --- | --- |
| Guangzhou (GZ) | 2 (Yuexiu, Liwan) /6 | 443 | 21 | 23 |
| Shenzhen (SZ) | 2 (Luohu, Nanshan) /10 | 85 | 21 | 1 |
| Dongguan | 2 (Nancheng, Wanjiang) /4 | 114 | 23 | 13 |
| Foshan (FS) | 2 (Gaoming, Sanshui) /5 | 189 | 28 | 14 |
| Zhongshan | 2 (Shiqi, Dongqu) /2 | 12 | 32 | 30 |
| Huizhou | 1 (Huicheng) /1 | 16 | 32 | 11 |
| Jiangmen | 2 (Jianghai, Pengjiang) /3 | 148 | 0 | 0 |
| Zhaoqing | 1 (Duanzhou) /1 | 29 | 3 | 0 |
| Zhuhai | 2 (Gaoxinqu, Hengqin) /4 | 87 | 9 | 0 |
| Jieyang^‡^ | 2 (Rongcheng, Jiedong) /2 | 19 | 9 | 0 |
| Shanwei^‡^ | 2 (Lufeng, Luhe) /4 | 78 | 0 | 0 |
| Meizhou^‡^ | 2 (Meijiang, Meixian) /2 | 44 | 0 | 20 |
| Zhanjiang^‡^ | 2 (Chikan, Xiashan) /3 | 99 | 0 | 0 |
| Yangjiang^‡^ | 1 (Jiangcheng) /1 | 26 | 1 | 0 |
| Yunfu^‡^ | 1 (Yuncheng) /1 | 60 | 2 | 2 |
| Sum-up | 26 | 1449 | 181 | 114 |

**^‡^**Non Pearl River Delta (PRD) region.

**^*^** The twenty-one prefectural-level cities from Guangdong province were stratified into cities locating in PRD region and non-PRD region. There were a total of nine cities in the PRD region and we enlisted all of the PRD cities in this study. For cities in non-PRD region, random number method was used to randomly select six cities out of the twelve non-PRD cities. A total of fifteen cities were randomly selected using the random number method. In choosing the cities that are in the Pearl River Delta region, no sampling bias emerged because we actually enlisted all those cities in the study. In sampling the cities that are not in the Pearl River Delta region, we adopted the random number method and randomly selected half of those cities. As for the sampling bias, the six randomly-selected cities are representative samples for non-Pearl-River-Delta cities because of the following reasons:

a) The six cities chosen were similar to other cities in the same region, in terms of H7N9 incidence. In the twelve cities of non-Pearl River Delta region, a total of seven (Chaozhou, Heyuan, Shantou, Shanwei, Jieyang, Meizhou, and Yangjiang) have had confirmed H7N9 cases during 2013-2015, and a total of five (Qingyuan, Maoming, Shaoguan, Yunfu and Zhanjiang) have not. And in fact, we sampled four cities (Shanwei, Jieyang, Meizhou, and Yangjiang) that have had confirmed H7N9 cases and two cities (Yunfu and Zhanjiang) that have not. Via the sampling procedures, representative samples were acquired. (detailed information on H7N9 incidence cases can be found on the official webpage of Health Department of Guangdong Province [1]).

b) The six cities chosen are distributed across the whole province in terms of geographical distribution.

c) Under the category of non-Pearl-River-Delta region [2], the six cities chosen are generally considered similar to those (other cities in the same region) that are not, in terms of demographics, economic development, etc.

In each city, live-poultry-restricted areas (geographical areas where trading of live poultry is restricted and chilled products are promoted) were randomly selected. For cities with only one live-poultry-restricted area, this live-poultry-restricted area was included. For cities having only two live-poultry-restricted areas, both were included, but for cities having more than two live-poultry-restricted areas, two were randomly selected using the random number method. The total number of live-poultry-restricted areas involved was 26. We used random number method to randomly select live-poultry markets and live-poultry farms at each live-poultry-restricted area in each enrolled city. A total of 52 live-poultry markets and 15 poultry farms were successfully enrolled in the study.

Consumers were approached at the live-poultry markets in Guangzhou, Foshan and Shenzhen. Consumers from other cities were surveyed online. The total number of consumers from the enrolled cites was 1449 (96.2%). All live-poultry workers either at the enrolled live-poultry markets or live-poultry farms were approached but 295 agreed to participate in the survey. We are able to estimate the rejection rate for live-poultry traders based on the number of live-poultry traders reported by Tan *et.al.* [3]. They have reported that there are no more than five live-poultry traders at each live-poultry market in the cities of Guangzhou, Shenzhen, Dongguan, Foshan and Yangjiang. Therefore, theoretically, we should have sampled a total number of 260 live-poultry traders from the 52 live-poultry markets selected. We then deduce, that we should have a rough rejection rate of 30.4% for live-poultry traders. The archived powers for the consumers, the live-poultry traders and the poultry farm workers were 93%, 93% and 95%, respectively. The power analysis was conducted in R package, version 3.3.2., and detailed description on power calculations can be found elsewhere [4].

^†^Thorough information on live-poultry-restricted areas for each city was obtained on the government website [5].

**Additional file 1: Table S3.** Attitudes towards avian influenza and preventive behaviours among the consumers and live-poultry worker^†^ from Guangdong, China, 2015.

| Attitudes and behaviours | Values | Sum-up  no. (%). | PRD^‡^  no. (%). | Non-PRD^‡‡^  no. (%). |
| --- | --- | --- | --- | --- |
| Consumers |  |  |  |  |
| Considering avian influenza to be serious | Yes | 1104 (76.2) | 853 (76.0) | 251 (77) |
|  | No | 299 (20.6) | 232 (20.7) | 67 (20.6) |
|  | No ideas | 46 (3.2) | 38 (3.4) | 8 (2.5) |
| Considering avian influenza to be preventable | Yes | 1341 (92.5) | 1040 (92.6) | 301 (92.3) |
|  | No | 47 (3.2) | 33 (2.9) | 14 (4.3) |
|  | No ideas | 61 (4.2) | 50 (4.5) | 11 (3.4) |
| Wearing a facemask upon contact with live poultry | Yes | 646 (44.6) | 504 (44.9) | 142 (43.6) |
|  | No | 803 (55.4) | 619 (55.1) | 184 (56.4) |
| Wearing gloves upon contact with live poultry | Yes | 682 (47.1) | 537 (47.8) | 145 (44.5) |
|  | No | 767 (52.9) | 586 (52.2) | 181 (55.5) |
| Handwashing after contact with live poultry | Yes | 1367 (94.3) | 1056 (94.0) | 311 (95.4) |
|  | No | 82 (5.7) | 67 (6.0) | 15 (4.6) |
| Acceptance of vaccination if available | Yes | 1140 (78.7) | 864 (76.9) | 276 (84.7) |
|  | No | 309 (21.3) | 259 (23.1) | 50 (15.3) |
| Live-poultry workers |  |  |  |  |
| Considering avian influenza to be serious | Yes | 26 (8.8) | 26 (10.0) | 0 (0.0) |
|  | No | 94 (31.9) | 90 (34.5) | 4 (11.8) |
|  | No ideas | 175 (59.3) | 145 (55.6) | 30 (88.2) |
| Considering avian influenza to be preventable | Yes | 35 (11.9) | 35 (13.4) | 0 (0.0) |
|  | No | 247 (83.7) | 214 (82) | 33 (97.1) |
|  | No ideas | 13 (4.4) | 12 (4.6) | 1 (2.9) |
| Wearing a facemask after contact with live poultry | Yes | 202 (68.5) | 198 (75.9) | 4 (11.8) |
|  | No | 93 (31.5) | 63 (24.1) | 30 (88.2) |
| Wearing gloves after contact with live poultry | Yes | 215 (72.9) | 207 (79.3) | 8 (23.5) |
|  | No | 80 (27.1) | 54 (20.7) | 26 (76.5) |
| Handwashing after contact with live poultry | Yes | 282 (95.6) | 248 (95.0) | 34 (100) |
|  | No | 13 (4.4) | 13 (5.0) | 0 (0.0) |

^†^Includes live-poultry traders and poultry farm workers.

^‡^PRD=Pearl river delta.

^‡‡^Non-PRD=Not pearl river delta.

**Additional file 1: Table S4.** Perception of the CSLPP^‡^ among consumers from Guangdong, China, 2015.

| Questionnaire Items | Value | Sum  N (%) | PRD  N (%) | Non-PRD  N (%) |
| --- | --- | --- | --- | --- |
| Heard of the central slaughtering policy | Yes | 847 (58.5) | 705 (62.8) | 142 (43.6) |
|  | No | 602 (41.5) | 418 (37.2) | 184 (56.4) |
| Knew the general working policy of the CSLPP | Yes | 760 (91.8) | 623 (90.8) | 137 (96.5) |
|  | No | 68 (8.2) | 63 (9.2) | 5 (3.5) |
| Knew the purpose of the CSLPP (to control avian influenza disease) | Yes | 808 (97.6) | 668 (97.4) | 140 (98.6) |
|  | No | 20 (2.4) | 18 (2.6) | 2 (1.4) |
| Which type of poultry product tastes the best | Live poultry | 649 (78.4) | 544 (79.3) | 105 (73.9) |
|  | Defeathered poultry | 85 (10.3) | 66 (9.6) | 19 (13.4) |
|  | Chilled poultry | 20 (2.4) | 15 (2.2) | 5 (3.5) |
|  | Frozen poultry | 2 (0.2) | 2 (0.3) | 0 (0.0) |
|  | No difference | 72 (8.7) | 59 (8.6) | 13 (9.2) |
| Poultry product to be of higher freshness/nutrition value | Live poultry | 599 (72.3) | 501 (73.0) | 98 (69.0) |
|  | Defeathered poultry | 78 (9.4) | 59 (8.6) | 19 (13.4) |
|  | Chilled poultry | 26 (3.1) | 22 (3.2) | 4 (2.8) |
|  | Frozen poultry | 2 (0.2) | 2 (0.3) | 0 (0.0) |
|  | No difference | 123 (14.9) | 102 (14.9) | 21 (14.8) |
| More convenient to buy chilled chicken than live ones | Yes | 659 (79.6) | 558 (81.3) | 101 (71.1) |
|  | No | 169 (20.4) | 128 (18.7) | 41 (28.9) |
| Influenced by median propaganda when purchasing chilled poultry product | Yes | 546 (65.9) | 460 (67.1) | 86 (60.6) |
|  | No | 282 (34.1) | 226 (32.9) | 56 (39.4) |
| Influenced by family preference when purchasing chilled poultry product | Yes | 563 (68.0) | 469 (68.4) | 94 (66.2) |
|  | No | 265 (32.0) | 217 (31.6) | 48 (33.8) |
| If a live poultry retail shop exists nearby | Yes | 502 (60.6) | 388 (56.6) | 114 (80.3) |
|  | No | 187 (22.6) | 172 (25.1) | 15 (10.6) |
|  | No idea | 139 (16.8) | 126 (18.4) | 13 (9.2) |
| Belief in food safety of chilled product | Yes | 529 (63.9) | 429 (62.5) | 100 (70.4) |
|  | No | 299 (36.1) | 257 (37.5) | 42 (29.6) |
| Belief in effectiveness of the CSLPP for prevention of avian influenza | Yes | 677 (81.8) | 568 (82.8) | 109 (76.8) |
|  | No | 151 (18.2) | 118 (17.2) | 33 (23.2) |
| Belief in effectiveness of the CSLPP for enhancement of environment | Yes | 716 (86.5) | 591 (86.2) | 125 (88.0) |
|  | No | 112 (13.5) | 95 (13.8) | 17 (12.0) |
| Frequency of purchase of poultry products after CSLPP | Increased | 66 (8.0) | 49 (7.1) | 17 (12.0) |
|  | Unchanged | 483 (58.3) | 394 (57.4) | 89 (62.7) |
|  | Decreased | 279 (33.7) | 243 (35.4) | 36 (25.4) |
| Which type of poultry product mostly purchased after CSLPP | Live poultry | 288 (34.8) | 223 (32.5) | 65 (45.8) |
|  | Defeathered poultry | 366 (44.2) | 308 (44.9) | 58 (40.8) |
|  | Chilled poultry | 168 (20.3) | 150 (21.9) | 18 (12.7) |
|  | Frozen poultry | 6 (0.7) | 5 (0.7) | 1 (0.7) |

^‡^CSLPP=Central slaughtering of live poultry policy.

**Additional file 1: Table S5.** Acceptability rate of the CSLPP among consumers, by cities, 2015.

| City where enrolled consumers lived | Support  N (%) | Disapproval  N (%) | Ignorance  N (%) |
| --- | --- | --- | --- |
| Guangzhou (GZ) | 122 (52.4) | 46 (19.7) | 65 (27.9) |
| Shenzhen (SZ) | 30 (68.2) | 4 (9.1) | 10 (22.7) |
| Dongguan | 40 (52.6) | 13 (17.1) | 23 (30.3) |
| Foshan (FS) | 77 (57.5) | 21 (15.7) | 36 (26.9) |
| Zhongshan | 8 (80.0) | 0 (0) | 2 (20.0) |
| Huizhou | 5 (38.5) | 1 (7.7) | 7 (53.8) |
| Jiangmen | 66 (53.7) | 16 (13.0) | 41 (33.3) |
| Zhaoqing | 15 (78.9) | 4 (21.1) | 0 (0.0) |
| Zhuhai | 25 (73.5) | 5 (14.7) | 4 (11.8) |
| Jieyang^‡^ | 3 (60.0) | 2 (40.0) | 0 (0.0) |
| Shanwei^‡^ | 14 (60.9) | 4 (17.4) | 5 (21.7) |
| Meizhou^‡^ | 16 (55.2) | 5 (17.2) | 8 (27.6) |
| Zhanjiang^‡^ | 12 (57.1) | 3 (14.3) | 6 (28.6) |
| Yangjiang^‡^ | 6 (37.5) | 8 (50.0) | 2 (12.5) |
| Yunfu^‡^ | 34 (70.8) | 4 (8.3) | 10 (20.8) |

**Additional file 1: Table S6.** Univariate logistics regression analysis on perception and acceptability rate of the CSLPP^‡^ among consumers.

| Variables | Values | Beta | Std.Err | *P* Value |
| --- | --- | --- | --- | --- |
| Influences on perception rate of the CSLPP | | | | |
| Gender | Male | -0.3 | 0.1 | 0.002^*^ |
| Age | ≤20 | -2.1 | 0.4 | <0.001^*^ |
|  | 21-30 | -1.2 | 0.4 | 0.002^*^ |
| Native-born Cantonese | Yes | 0.8 | 0.1 | <0.001^*^ |
| Score on knowledge of avian influenza | Class One | -0.6 | 0.3 | 0.027^*^ |
|  | Class Two | -0.3 | 0.1 | 0.007^*^ |
| Considering avian influenza to be a preventable disease | Yes | 0.7 | 0.3 | 0.009^*^ |
| Free-will to accept avian influenza vaccination if available | Yes | -0.3 | 0.1 | 0.024^*^ |
| Influence on acceptability rate of the CSLPP among those who were unfamiliar with the policy | | | | |
| Income (Yuan per capita/month) |  |  |  |  |
|  | 1001-1999 | -0.9 | 0.4 | 0.013^*^ |
|  | 2000-2999 | -0.1 | 0.4 | 0.845 |
|  | 3000-3999 | 0.0 | 0.4 | 0.928 |
|  | 4000-4999 | -0.2 | 0.4 | 0.692 |
|  | 5000-5999 | -0.3 | 0.3 | 0.405 |
| Native-born Cantonese | Yes | -0.4 | 0.2 | 0.016^*^ |
| Preference for poultry slaughtered | On site | 1.4 | 0.7 | 0.036^*^ |
|  | At home | 0.8 | 0.7 | 0.261 |
| Fever induced by infection of avian influenza | No | 1.6 | 0.9 | 0.075 |
|  | Yes | 2.3 | 0.8 | 0.005^*^ |
| Wearing a facemask upon contact with live poultry | Yes | 0.6 | 0.2 | <0.001^*^ |
| Wearing gloves upon contact with live poultry | Yes | 0.6 | 0.2 | <0.001^*^ |
| Free-will to accept avian influenza vaccination if available | Yes | 0.4 | 0.2 | 0.043^*^ |
| Influence on acceptability rate of the CSLPP among those who were familiar with the policy | | | | |
| Native-born Cantonese | Yes | -0.5 | 0.2 | 0.009^*^ |
| Preference of purchasing live poultry | Yes | -0.9 | 0.2 | <0.001^*^ |
| Poultry product to be of higher freshness/ nutrition value | Chilled | -12.2 | 0.4 | <0.001^*^ |
|  | Defeathered | -12.0 | 0.2 | <0.001^*^ |
|  | Live | -13.1 | 0.1 | <0.001^*^ |
|  | No difference | -12.4 | 0.2 | <0.001^*^ |
| More convenient to buy chilled chicken than live ones | Yes | 1.7 | 0.2 | <0.001^*^ |
| Influenced by median propaganda | Yes | 0.7 | 0.1 | <0.001^*^ |
| Family preference | Yes | 0.4 | 0.1 | 0.012^*^ |
| If a live-poultry retail shop exists nearby | No | 0.5 | 0.2 | 0.018^*^ |
|  | Yes | 0.4 | 0.2 | 0.017^*^ |
| Belief in food safety of chilled product | Yes | 1.0 | 0.1 | <0.001^*^ |
| Belief in effectiveness of the CSLPP | For prevention of avian influenza | 2.3 | 0.2 | <0.001^*^ |
|  | For enhancement of environment | 2.7 | 0.2 | <0.001^*^ |
| Frequency of purchase of poultry products after CSLPP | Decreased | -0.7 | 0.1 | <0.001^*^ |
|  | Increased | 1.5 | 0.4 | <0.001^*^ |
| Fever | No ideas | 1.0 | 0.8 | 0.224 |
|  | Yes | 1.8 | 0.8 | 0.022^*^ |
| Direct transmission to others | No ideas | -0.7 | 0.3 | 0.026^*^ |
|  | Yes | 0.2 | 0.2 | 0.399 |
| Considering avian influenza disease to be a serious one | No | 0.7 | 0.4 | 0.047^*^ |
|  | Yes | 1.5 | 0.3 | <0.001^*^ |
| Considering avian influenza disease to be a preventable one | Yes | 1.2 | 0.4 | 0.001^*^ |
| Wearing a facemask upon contact with live poultry | Yes | 0.5 | 0.1 | <0.001^*^ |
| Wearing gloves upon contact with live poultry | Yes | 0.6 | 0.1 | <0.001^*^ |
| Handwashing after contact with live poultry | Yes | 0.8 | 0.3 | 0.009^*^ |
| Free-will to accept avian influenza vaccination if available | Yes | 0.5 | 0.2 | 0.002^*^ |

*Indicating a statistical significance (*P* < 0.05).

^‡^CSLPP=Central slaughtering of live poultry policy.

**Additional file 1: Table S7.** Univariate analysis of factors influencing the support of the CSLPP^‡^ among sub-groups of live-poultry workers.

| Variables | Values | Beta | Std. Error^#^ | *P* Value | OR | 95% CI for OR | |
| --- | --- | --- | --- | --- | --- | --- | --- |
|  |  |  |  |  |  | 2.5% | 97.5% |
| Live-poultry traders |  |  |  |  |  |  |  |
| Gender | Male | -0.25 | 0.29 | 0.387 | 0.78 | 0.44 | 1.37 |
| Age | ≤30 | -0.05 | 0.55 | 0.927 | 0.95 | 0.32 | 2.80 |
|  | 31-40 | 0.38 | 0.46 | 0.406 | 1.46 | 0.60 | 3.60 |
|  | 41-50 | -0.19 | 0.42 | 0.653 | 0.83 | 0.36 | 1.89 |
| Employment status | Employer | -0.73 | 0.31 | 0.020^*^ | 0.48 | 0.26 | 0.89 |
| Region | PRD | 0.89 | 0.71 | 0.210 | 2.44 | 0.65 | 11.7 |
| Drop of trading volume after CSLPP | Yes | -2.10 | 0.53 | <0.001^*^ | 0.12 | 0.04 | 0.32 |
| Rise of cost after CSLPP | Yes | -0.13 | 0.34 | 0.692 | 0.87 | 0.45 | 1.70 |
| Profit change | Decrease | -2.02 | 1.14 | 0.076 | 0.13 | 0.01 | 0.94 |
|  | Unchanged | -0.59 | 1.20 | 0.622 | 0.55 | 0.03 | 4.60 |
| Subsidy given | Yes | -0.82 | 0.29 | 0.005^*^ | 0.44 | 0.25 | 0.78 |
| Moderate subsidy | Yes | 1.41 | 0.42 | 0.001^*^ | 4.09 | 1.83 | 9.45 |
| Policy explained by market manager | Yes | 0.62 | 0.53 | 0.247 | 1.86 | 0.67 | 5.65 |
| Belief in effectiveness of the CSLPP | For prevention of avian influenza | 2.84 | 0.39 | <0.001^*^ | 17.14 | 8.20 | 38.65 |
|  | For betterment of environment | 2.26 | 0.51 | <0.001^*^ | 9.63 | 3.81 | 29.61 |
| Inconvenience to work | Yes | -1.24 | 0.32 | <0.001^*^ | 0.29 | 0.15 | 0.54 |
| Convenience to work | Yes | 1.23 | 0.33 | <0.001^*^ | 3.43 | 1.82 | 6.60 |
| Score on knowledge of avian influenza | Class One | -2.14 | 0.40 | <0.001^*^ | 0.12 | 0.05 | 0.25 |
|  | Class Two | -1.34 | 0.35 | <0.001^*^ | 0.26 | 0.13 | 0.52 |
| Belief in avian influenza to be serious | Yes | 0.86 | 0.49 | 0.078 | 2.37 | 0.92 | 6.38 |
|  | No | 0.06 | 0.48 | 0.895 | 1.07 | 0.42 | 2.81 |
| Belief in avian influenza to be preventable | No idea | -1.38 | 0.41 | 0.001^*^ | 0.25 | -2.21 | -0.58 |
|  | No | 0.20 | 0.59 | 0.742 | 1.22 | -0.96 | 1.40 |
| Living in GZ,SZ,FS | Yes | 0.86 | 0.30 | 0.003^*^ | 2.37 | 1.33 | 4.25 |
| Poultry farm workers |  |  |  |  |  |  |  |
| Gender | Male | -0.09 | 0.45 | 0.837 | 0.91 | 0.36 | 2.19 |
| Age | 31-40 | 0.52 | 0.74 | 0.481 | 1.68 | 0.39 | 7.27 |
|  | 41-50 | 1.14 | 0.68 | 0.094 | 3.14 | 0.81 | 12.18 |
|  | 51-60 | 0.57 | 0.65 | 0.388 | 1.76 | 0.47 | 6.36 |
| Employment status | Employer | 0.88 | 0.82 | 0.284 | 2.41 | 0.56 | 16.58 |
| Region | PRD | -2.93 | 1.05 | 0.005^*^ | 0.05 | 0.00 | 0.28 |
| Business history (years) | 6-10 | -0.20 | 0.50 | 0.681 | 0.82 | 0.31 | 2.17 |
|  | 11~20 | 0.86 | 0.74 | 0.244 | 2.37 | 0.60 | 11.93 |
|  | 21-50 | 1.40 | 0.64 | 0.028 | 4.06 | 1.25 | 16.01 |
| Farm scale | Small size | 0.69 | 0.58 | 0.235 | 1.99 | 0.66 | 6.59 |
|  | Medium size | 0.80 | 0.46 | 0.085 | 2.22 | 0.90 | 5.63 |
| Drop of trading volume | Yes | -2.72 | 0.65 | <0.001^*^ | 0.07 | 0.01 | 0.21 |
| Profit change | Unchanged | 0.65 | 1.23 | 0.598 | 1.92 | 0.09 | 17.99 |
|  | Decrease | -2.30 | 1.11 | 0.038^*^ | 0.10 | 0.01 | 0.63 |
| Policy explained by government workers | Yes | 1.61 | 0.47 | 0.001^*^ | 5.01 | 2.02 | 12.78 |
| Belief in effectiveness of the CSLPP | For prevention of avian influenza | 2.55 | 0.57 | <0.001^*^ | 12.75 | 4.31 | 41.65 |
|  | For enhancement of environment | 2.55 | 0.57 | <0.001^*^ | 12.75 | 4.31 | 41.65 |
| Inconvenience to work due to the policy | Yes | -2.04 | 0.48 | <0.001^*^ | 0.13 | 0.05 | 0.32 |
| Convenience to work due to the policy | Yes | 2.55 | 0.59 | <0.001^*^ | 12.80 | 4.45 | 46.87 |
| Score on knowledge of avian influenza | Class One | -1.71 | 0.77 | 0.025^*^ | 0.18 | 0.04 | 0.79 |
|  | Class Two | -0.57 | 0.57 | 0.314 | 0.56 | 0.17 | 1.63 |
| Avian influenza to be a serious disease | Yes | 1.24 | 0.89 | 0.164 | 3.45 | 0.60 | 20.71 |
|  | No | 1.46 | 0.81 | 0.073 | 4.29 | 0.85 | 22.06 |
| Avian influenza to be a preventable disease | Yes | 2.17 | 1.02 | 0.034^*^ | 8.73 | 1.18 | 78.35 |
|  | No | -0.44 | 1.62 | 0.787 | 0.65 | 0.02 | 15.72 |

^*^Indicating a statistical significance (*P* < 0.05).

-Indicates not available for calculation.

^#^Std.Error=Standard deviation of error.

^‡^CSLPP=Central slaughtering of live poultry policy.

^¶^Classification of score on knowledge of avian influenza: a) Class One: scoring zero to four; b) Class Two: scoring five to eight; c) Class Three: scoring nine or above.

**Additional file 1: Q1.** Questionnaire targeting consumers.

Survey Consent Form

Dear Sir/Ms,

We are investigators recruited by School of Public Health, Sun Yat-sen University. The survey involves answering some general demographics questions and some questions about your knowledge and attitude towards central slaughtering policy and avian influenza. The survey takes about 10 minutes to complete. The purpose of the survey is to help assess your acceptability of the policy in Guangdong for further review of the implementation of the policy. Your participation is completely voluntary, and your responses will be completely anonymous. The data I collect will be analysed at the group level only. You do not have to answer any question you would rather not answer. There are no consequences if you decide not to complete the survey.

If you agree to complete the survey (JUST TICK the proper answer under the choice), please do NOT write your name on it. After you finish filling it out, please put the survey in the envelope and then place the envelope in the box provided. By filling out the survey you are consenting to participate.

If you do not want to complete the survey, just return the blank form and envelope to me now.

If you have any questions about the assignment or your rights as a volunteer participant in this subject, you can reach me at 158-7532-7398. This project has been approved by the Institutional Review Board of the Sun Yat-sen University, College of Public Health (Reference Number: L2016-045).

Thank you for your participation.

Basic Information

| A1.What is your gender? ①Male ②Female |
| --- |
| A2.Age___? |
| A3.Level of education?①Primary or below ②Secondary ③High School ④Tertiary or above |
| A4.Family monthly Income per capita? ①1000 or below ②1001-1999 ③2000-2999  ④3000-3999 ⑤4000-4999 ⑥5000-5999 ⑦6000 and above |
| A5.Family Address? _________CITY__________DISTRICT（COUNTY） |
| A6.Whether or not a local Cantonese? ①Yes ②No |
| A7.Do you or your family have past experience of purchasing live poultry? ①Yes ②No |
| A8.Who will slaughter the live poultry for you after purchase? ①poultry traders ②Family member(s) ③By yourself |

Perception of central slaughtering policy

| B1. Are you aware of central slaughtering policy？①Yes（proceed to B3） ②No（proceed to B2 then to D1） |
| --- |
| B2.Will you support the central slaughtering policy？(proceed to D1) ①Yes ②No |
| B3.Do you know the content of the policy? ①Yes ②No |
| B4.Do you know the aim of the policy is to control avian influenza? ①Yes ②No |
| B5. Where do you learn about the policy? (Multiple-choices) ①TV, broadcast ②Online ③Newspaper, journals ④Friends around ⑤Bulletins boards ⑥other____ |

Acceptability of central slaughtering policy

| C1.Which type of poultry do you think tastes better? ①live poultry ②defeathered poultry ③chilled poultry ④frozen poultry ⑤does not matter |
| --- |
| C2.Which type of poultry do you think contains more nutrition? ①live poultry ②defeathered poultry ③chilled poultry ④frozen poultry ⑤does not matter |
| C3.Do you think it more convenient to buy chilled products than live ones? ①Yes ②No |
| C4.Will media propaganda influence your choice of purchase? ①Yes ②No |
| C5. Will family’s opinions influence your choice of purchase? ①Yes ②No |
| C6. Is there a live-poultry retailer nearby your home? ①Yes ②No ③No ideas |
| C7. Do you believe in the safety of the chilled products? ①Yes ②No |
| C8.Do you believe in the effectiveness of the policy in control avian influenza? ①Yes ②No |
| C9.Do you believe in the betterment of the environment as a result of the policy? ①Yes ②No |
| C10.What’s your frequency of buying poultry after the launch of the policy? ①Increased  ②Unchanged ③Decreased |
| C11.Which type of poultry do you temp to buy after the launch of the policy? ①live poultry  ②defeathered poultry ③chilled poultry ④frozen poultry |
| C12.Do you support the policy? ①Yes ②No |

Knowledge, perceptions and behaviour of avian influenza

| D1.Will one get a fever if infected with avian influenza? ①Yes ②No ③No ideas |
| --- |
| D2. Will one get a pneumonia if infected with avian influenza? ①Yes ②No ③No ideas |
| D3. Can avian influenza spread between individuals? ①Yes ②No ③No ideas |
| D4. Will one die if infected with avian influenza? ①Yes ②No ③No ideas |
| D5.What type(s) of animals do you think can help the spread of avian influenza? (Multiple-choices) ①domestic poultry  ②wild birds ③Pigs or other animals |
| D6.Will you be infected if in contact with healthy poultry? ①Yes ②No ③No ideas |
| D7. Will you be infected if in contact with diseased poultry? ①Yes ②No ③No ideas |
| D8. Will you be infected if in contact with dead poultry? ①Yes ②No ③No ideas |
| D9.Can you prevent the spread of avian influenzawhen food is cooked thoroughly？①Yes ②No ③No ideas |
| D10.Where do you learn about avian influenza? (Multiple-choices) ①TV, broadcast ②Online ③Newspaper, journals ④Friends around ⑤Bulletins boards ⑥other____ |
| D11.Is there a past confirmed case reported in your city? ①Yes ②No ③No ideas |
| D12.Do you think avian influenza as a serious disease? ①Yes ②No ③No ideas |
| D13.Do you think avian influenza as a preventable disease? ①Yes ②No ③No ideas |
| D14.Do you wear facemask when in contact with live poultry? ①Yes ②No |
| D15. Do you wear hand gloves when in contact with live poultry?①Yes ②No |
| D16. Do you wash your hands after contact with live poultry?①Yes ②No |
| D17.Will you choose to take a vaccine for yourself if available? ①Yes ②No |

You have finished the survey. Thank you for your participation!

**Additional file 1: Q2.** Questionnaire targeting live-poultry traders.

Survey Consent Form

Dear Sir/Ms,

We are investigators recruited by School of Public Health, Sun Yat-sen University. The survey involves answering some general demographics questions and some questions about your knowledge and attitude towards central slaughtering policy and avian influenza. The survey takes about 10 minutes to complete. The purpose of the survey is to help assess your acceptability of the policy in Guangdong for further review of the implementation of the policy. Your participation is completely voluntary, and your responses will be completely anonymous. The data I collect will be analysed at the group level only. You do not have to answer any question you would rather not answer. There are no consequences if you decide not to complete the survey.

If you agree to complete the survey (JUST TICK the proper answer under the choice), please do NOT write your name on it. After you finish filling it out, please put the survey in the envelope and then place the envelope in the box provided. By filling out the survey you are consenting to participate.

If you do not want to complete the survey, just return the blank form and envelope to me now.

If you have any questions about the assignment or your rights as a volunteer participant in this subject, you can reach me at 158-7532-7398. This project has been approved by the Institutional Review Board of the Sun Yat-sen University, College of Public Health (Reference Number: L2016-045).

Thank you for your participation.

Basic Information

| A1.What is your gender? ①Male ②Female |
| --- |
| A2.Age___? |
| A3.Working Address? _________CITY__________DISTRICT（COUNTY） |
| A4.Employment status? ①Employer ②Employee |
| A5.What type(s) of chilled poultry products do you sell? ①Chicken ②Ducks ③Geese ④Pigeons⑤Quails ⑥Other |
| A6.What’s your sale volume per day? _____No./Day |

Perception of central slaughtering policy

| B1. Are you aware of central slaughtering policy？①Yes（proceed to B3） ②No（proceed to B2 then to D1） |
| --- |
| B2.Will you support the central slaughtering policy？(proceed to D1) ①Yes ②No |
| B3.Do you know the content of the policy? ①Yes ②No |
| B4.Do you know the aim of the policy is to control avian influenza? ①Yes ②No |
| B5. Where do you learn about the policy? (Multiple-choices) ①TV, broadcast ②Online ③Newspaper, journals ④Friends around ⑤Market managers ⑥other____ |

Acceptability of central slaughtering policy

| C1.Have you experienced a drop of sale after the implementation of central slaughtering policy?   1. Yes (proceed next question) ②No (proceed to C3) |
| --- |
| C2.What’s the probability of your drop of sale? ①≤10% ②20-30% ③40-50% ④>50% |
| C3. Have you experienced a rise of cost after the implementation of central slaughtering policy?   1. Yes ②No |
| C4. What’s the probability of your rise of cost? ①≤10% ②20-30% ③40-50% ④>50% |
| C5. Any change of profit after the implementation of central slaughtering policy? ①Increased   1. Unchanged ③Decreased |
| C6. Is there any subsidy given by the local government? ①Yes (proceed) ②No (jump to C8) |
| C7. Do you think the subsidy sufficient? ①Yes ②No |
| C8.Have the market managers explained everything about the policy? ①Yes ②No |
| C9.Do you believe in the effectiveness of the policy in control avian influenza? ①Yes ②No |
| C10. Do you believe in the betterment of the environment as a result of the policy? ①Yes ②No |
| C11.Have you experienced inconvenience brought by the policy?   1. Yes______(and write down what they are) ②No |
| C12.Have you experienced convenience brought by the policy?   1. Yes______(and write down what they are) ②No |
| C13.Do you support the policy? ①Yes ②No |

Knowledge, perceptions and behaviour of avian influenza

| D1.Will one get a fever if infected with avian influenza? ①Yes ②No ③No ideas |
| --- |
| D2. Will one get a pneumonia if infected with avian influenza? ①Yes ②No ③No ideas |
| D3. Can avian influenza spread between individuals? ①Yes ②No ③No ideas |
| D4. Will one die if infected with avian influenza? ①Yes ②No ③No ideas |
| D5.What type(s) of animals do you think can help the spread of avian influenza? (Multiple-choices) ①domestic poultry  ②wild birds ③Pigs or other animals |
| D6.Will you be infected if in contact with healthy poultry? ①Yes ②No ③No ideas |
| D7. Will you be infected if in contact with diseased poultry? ①Yes ②No ③No ideas |
| D8. Will you be infected if in contact with dead poultry? ①Yes ②No ③No ideas |
| D9.Can you prevent the spread of avian influenzawhen food is cooked thoroughly？①Yes ②No ③No ideas |
| D10.Where do you learn about avian influenza? (Multiple-choices) ①TV, broadcast ②Online ③Newspaper, journals ④Friends around ⑤Bulletins boards ⑥other____ |
| D11.Is there a past confirmed case reported in your city? ①Yes ②No ③No ideas |
| D12.Do you think avian influenza as a serious disease? ①Yes ②No ③No ideas |
| D13.Do you think avian influenza as a preventable disease? ①Yes ②No ③No ideas |
| D14.Do you wear facemask when in contact with live poultry? ①Yes ②No |
| D15.Which type of facemask do you use? ①Normal facemask ②Medical facemask |
| D16.Will you discard the facemask after use? ①Yes ②No |
| D17. Do you wear hand gloves when in contact with live poultry?①Yes ②No |
| D18. Do you wash your hands after contact with live poultry?①Yes ②No |
| D19. Will you change your clothes immediately after contact with live poultry?①Yes ②No |
| D20.Will you choose to take a vaccine for yourself if available? ①Yes ②No |

You have finished the survey. Thank you for your participation!

**Additional file 1: Q3.** Questionnaire targeting poultry farm workers.

Survey Consent Form

Dear Sir/Ms,

We are investigators recruited by School of Public Health, Sun Yat-sen University. The survey involves answering some general demographics questions and some questions about your knowledge and attitude towards central slaughtering policy and avian influenza. The survey takes about 10 minutes to complete. The purpose of the survey is to help assess your acceptability of the policy in Guangdong for further review of the implementation of the policy. Your participation is completely voluntary, and your responses will be completely anonymous. The data I collect will be analysed at the group level only. You do not have to answer any question you would rather not answer. There are no consequences if you decide not to complete the survey.

If you agree to complete the survey (JUST TICK the proper answer under the choice), please do NOT write your name on it. After you finish filling it out, please put the survey in the envelope and then place the envelope in the box provided. By filling out the survey you are consenting to participate.

If you do not want to complete the survey, just return the blank form and envelope to me now.

If you have any questions about the assignment or your rights as a volunteer participant in this subject, you can reach me at 158-7532-7398. This project has been approved by the Institutional Review Board of the Sun Yat-sen University, College of Public Health (Reference Number: L2016-045).

Thank you for your participation.

Basic Information

| A1.What is your gender? ①Male ②Female |
| --- |
| A2.Age___? |
| A3.Working Address? _________CITY__________DISTRICT（COUNTY） |
| A4.Employment status? ①Employer ②Employee |
| A5.What type(s) of poultry do you breed? ①Chicken ②Ducks ③Geese ④Pigeons⑤Quails ⑥Other |
| A6.What’s your farm’s history of business? _____(years) |

Perception of central slaughtering policy

| B1. Are you aware of central slaughtering policy？  ①Yes（proceed to B3） ②No（proceed to B2 then to D1） |
| --- |
| B2.Will you support the central slaughtering policy？(proceed to D1) ①Yes ②No |
| B3.Do you know the content of the policy? ①Yes ②No |
| B4.Do you know the aim of the policy is to control avian influenza? ①Yes ②No |
| B5. Where do you learn about the policy? (Multiple-choices) ①TV, broadcast ②Online ③Newspaper, journals ④Friends around ⑤Market managers ⑥other____ |

Acceptability of central slaughtering policy

| C1.Have you experienced a drop of sale after the implementation of central slaughtering policy?   1. Yes (proceed next question) ②No (proceed to C3) |
| --- |
| C2.What’s the probability of your drop of sale? ①≤10% ②20-30% ③40-50% ④>50% |
| C3. Any change of profit after the implementation of central slaughtering policy?  ①Increased   1. Unchanged ③Decreased |
| C4.Have the government workers explained everything about the policy to you?   1. Yes ②No |
| C5.Do you believe in the effectiveness of the policy in control avian influenza?   1. Yes ②No |
| C6. Do you believe in the betterment of the environment as a result of the policy?  ①Yes ②No |
| C7.Have you experienced inconvenience brought by the policy?   1. Yes______(and write down what they are) ②No |
| C8.Have you experienced convenience brought by the policy?   1. Yes______(and write down what they are) ②No |
| C9.Do you support the policy? ①Yes ②No |

Knowledge, perceptions and behaviour of avian influenza

| D1.Will one get a fever if infected with avian influenza? ①Yes ②No ③No ideas |
| --- |
| D2. Will one get a pneumonia if infected with avian influenza? ①Yes ②No ③No ideas |
| D3. Can avian influenza spread between individuals? ①Yes ②No ③No ideas |
| D4. Will one die if infected with avian influenza? ①Yes ②No ③No ideas |
| D5.What type(s) of animals do you think can help the spread of avian influenza? (Multiple-choices) ①domestic poultry  ②wild birds ③Pigs or other animals |
| D6.Will you be infected if in contact with healthy poultry? ①Yes ②No③No ideas |
| D7. Will you be infected if in contact with diseased poultry? ①Yes②No③No ideas |
| D8. Will you be infected if in contact with dead poultry? ①Yes ②No③No ideas |
| D9.Can you prevent the spread of avian influenzawhen food is cooked thoroughly？①Yes   1. No ③No ideas |
| D10.Where do you learn about avian influenza? (Multiple-choices)  ①TV, broadcast ②Online③Newspaper, journals ④Friends around ⑤Bulletins boards ⑥other____ |
| D11.Is there a past confirmed case reported in your city? ①Yes ②No ③No ideas |
| D12.Do you think avian influenza as a serious disease? ①Yes ②No ③No ideas |
| D13.Do you think avian influenza as a preventable disease? ①Yes ②No ③No ideas |
| D14.Do you wear facemask when in contact with live poultry? ①Yes ②No |
| D15.Which type of facemask do you use? ①Normal facemask  ②Medical facemask |
| D16.Will you discard the facemask after use? ①Yes ②No |
| D17. Do you wear hand gloves when in contact with live poultry?①Yes ②No |
| D18. Do you wash your hands after contact with live poultry?①Yes ②No |
| D19. Will you change your clothes immediately after contact with live poultry?   1. Yes ②No |
| D20.Will you vaccinate your poultry to prevent avian influenza? ①Yes ②No |
| D21.Will you choose to take a vaccine for yourself if available? ①Yes ②No |

You have finished the survey. Thank you for your participation!

**References:**

1. The Health Department of Guangdong Province. 2017. http://www.gdwst.gov.cn/. Accessed 14 Mar 2017.

2. A surveillance study on economic development of different regions in Guangdong Province. 2017. http://www.gdstats.gov.cn/jyky/tjky/kycg/201507/t20150721_309887.html. Accessed 14 Mar 2017.

3. Xiaohua T, Limei S, Jianfeng H, Haojie Z, Wentao L, Zhongxian C, Min K, Jinyan L. Cross-sectional study on management measures of live poultry market in Guangdong province. South China J Prev Med 2015, **41**(01):61-64.

4. Hsieh FY, Bloch DA, Larsen MD. A simple method of sample size calculation for linear and logistic regression. STAT MED 1998, **17**(14):1623-1634.

5. Guangdong Food and Drug Administration--Announcement on Live Poultry Restricted Areas. 2016. http://www.gdda.gov.cn/publicfiles/business/htmlfiles/jsjzz/pgeshidongtai/201512/315337.htm. Accessed 22 Feb 2016.
